# Polymorphic DNA microsatellite markers for forensic individual identification and parentage analyses of seven threatened species of parrots (family Psittacidae)

**DOI:** 10.7717/peerj.2416

**Published:** 2016-09-22

**Authors:** Catherine Jan, Luca Fumagalli

**Affiliations:** 1 Unité de Génétique Forensique, Centre Universitaire Romand de Médecine Légale, Chemin de la Vulliette 4, CH-1000, Lausanne 25, Switzerland; 2 Laboratory for Conservation Biology, Department of Ecology and Evolution, Biophore, University of Lausanne, CH-1015, Lausanne, Switzerland

**Keywords:** Genetic diversity, Conservation, Microsatellites, Red-browed amazon, Yellow-headed amazon, Red-fronted macaw, Lear’s macaw, Red-tailed amazon, Blue-headed macaw, STR markers, Red-spectacled amazon

## Abstract

The parrot family represents one of the bird group with the largest number of endangered species, as a result of habitat destruction and illegal trade. This illicit traffic involves the smuggling of eggs and animals, and the laundering through captive breeding facilities of wild-caught animals. Despite the huge potential of wildlife DNA forensics to determine with conclusive evidence illegal trade, current usage of DNA profiling approaches in parrots has been limited by the lack of suitable molecular markers specifically developed for the focal species and by low cross-species polymorphism. In this study, we isolated DNA microsatellite markers in seven parrot species threatened with extinction (*Amazona brasiliensis*, *A. oratrix*, *A. pretrei*, *A. rhodocorytha*, *Anodorhynchus leari*, *Ara rubrogenys* and *Primolius couloni*). From an enriched genomic library followed by 454 pyrosequencing, we characterized a total of 106 polymorphic microsatellite markers (mostly tetranucleotides) in the seven species and tested them across an average number of 19 individuals per species. The mean number of alleles per species and across loci varied from 6.4 to 8.3, with the mean observed heterozygosities ranging from 0.65 to 0.84. Identity and parentage exclusion probabilities were highly discriminatory. The high variability displayed by these microsatellite loci demonstrates their potential utility to perform individual genotyping and parentage analyses, in order to develop a DNA testing framework to determine illegal traffic in these threatened species.

## Introduction

Poaching, i.e. illegal harvesting, trade, transport, possession and use of wildlife, is among the most serious threats to the persistence of many endangered species, and represents in profits one of the world’s largest illegal trafficking (Haken, 2011; South & Wyatt, 2011). Wildlife DNA forensics deals with the genetic identification of illegal trade in endangered animal and plant species. Because of powerful and unambiguous identification, and the possibility to use very small (and even degraded) DNA samples from various sources (e.g. tissue, scats, hair, feathers, eggshells), molecular techniques have become increasingly important in the detection of illegal hunting and traffic of wildlife. This relatively recent discipline is now becoming a key investigative tool to fight wildlife crime and assist law enforcement and wildlife management agencies (reviewed in Iyengar (2014), Johnson, Wilson-Wilde & Linacre (2014) and Ogden, Dawnay & McEwing, (2010)).

Wild birds in general and Neotropical parrots (family Psittacidae) in particular are highly prized by collectors for their colourful plumage, mimicry ability, exotic appeal and rarity. The Psittacidae family contain over 370 species worldwide, including parrots, macaws, amazons, cockatoos (Collar, 1997). Habitat loss in combination with illegal trade are a significant threat to these species. According to the IUCN Red List of Threatened Species, 101 of the world’s parrot species are considered to be threatened with extinction (International Union for Conservation of Nature and Natural Resources, 2015). Almost all of the world’s parrot species are protected by the Convention on International Trade in Endangered Species of Wild Fauna and Flora (CITES), with over 40 species being listed on CITES Appendix I, the Convention’s highest level of protection. All other parrots (excluding three species) are listed in Appendix II, which regulates commercial trade through a permitting system (UNEP-WCMC, 2015). The illicit trade in parrots involves both the smuggling of eggs and live animals from the wild and the falsification of documents.

Captive breeding is one of the primary activities for the production of animals of commercial value, in particular for many endangered parrot species. This creates the possibility that individuals are taken from the wild illegally and laundered through captive breeding facilities by claiming them to be legitimate progeny of already captive individuals. Parentage verification using high-resolution markers such as DNA microsatellites can establish without ambiguity whether a claim of parentage is true, and therefore provide the ultimate evidence of illegal activity.

Despite the potential of wildlife DNA forensics to determine with conclusive evidence illegal trade or harvest from the wild, the implementation of such analyses in parrots has been very limited to date for different reasons. First, the number of genetic markers (i.e. microsatellites) specifically developed for the species of interest is low, constraining the use of markers developed in other species (cross-amplification), with a frequent loss in marker specificity and resolution, and therefore loss of statistical power (Presti & Wasko, 2014). Second, success rate in isolating microsatellites from parrots has always been reported as low (Hughes, Melland & Beissinger, 1998). Third, because of the extreme rarity and therefore difficulty of sampling some species, compiling population DNA databases for the computation of the statistical certainty of a parental assignment is a very difficult and challenging task.

Here we report the development and characterization of specific, polymorphic microsatellite loci for seven parrot species which are currently involved in local and international illegal traffic for pet supply, all being threatened with extinction according to the IUCN criteria (International Union for Conservation of Nature and Natural Resources, 2015) and CITES-listed in Appendix I. These species comprise the Red-tailed amazon (*Amazona brasiliensis*), the Yellow-headed amazon (*A. oratrix*), the Red-spectacled amazon (*A. pretrei*), the Red-browed amazon (*A. rhodocorytha*), the Lear’s macaw (*Anodorhynchus leari*), the Red-fronted macaw (*Ara rubrogenys*) and the Blue-headed macaw (*Primolius couloni*). These markers will allow a genetic identification and parentage DNA testing framework to be set up to assist the detection of illegal trade and traffic of these endangered parrot species.

## Materials and Methods

Total genomic DNA was isolated from blood samples using one individual per species with the DNeasy Blood & Tissue Kit (Qiagen). To isolate the microsatellite sequences from the genomic DNA extractions, an hybridization enrichment protocol with SSR oligonucleotides (for GATA/GTAT motifs) followed by 454-pyrosequencing was performed by a private company (Ecogenics GmbH, Balgach, Switzerland). The same company designed a list of suitable primer pairs for the seven species with a filtering threshold of 350 base pairs.

Blood and feather samples were collected from private and public aviaries in several European countries (see Acknowledgements). DNA extractions were performed using the QIAamp DNA Mini Kit (Qiagen) following the manufacturers’ instructions with some modifications. The samples were digested in 180 μL ATL buffer and 20–40 μL proteinase K, then incubated overnight at 56 °C using a thermomixer. The lysate was then loaded through a QIAshredder homogenizing column (Qiagen) and centrifuged for 5 min at 14,000 rpm. All further steps were performed according to the QIAamp kit protocol. In the final step, DNA was eluted in 120–200 μL elution buffer and stored at −20 °C.

We tested for amplification and polymorphism about 50 primer pairs per species in a panel of about five individuals per species. PCR reactions were performed in a final volume of 21 μL, containing 1 μL of DNA template, 1 unit AmpliTaq Gold (Applied Biosystems), 1 × PCR II buffer, 3.5 mM MgCl_2_, 0.1 μM each primer, 0.2 mM each dNTPs, 0.2 mg/ml BSA (Sigma). PCRs were run as follows: an initial denaturation for 10 min at 95 °C, followed by 35 cycles of 30 s at 95 °C, 30 s at 57 °C, 45 s at 72 °C, and final elongation for 30 min at 72 °C.

The forward primer of each primer set was fluorescently labelled with either 6FAM, ATTO532, ATTO550 and ATTO565 (Table S1). Fragment analysis was conducted on an ABI 3130 automated sequencer (Applied Biosystems). Alleles were scored using GeneMapper v3.2.1 (Applied Biosystems).

The number of alleles per locus (*A*), observed (*H*_O_) and expected (*H*_E_) heterozygosities, identity (*P*_ID_ and *P*_IDsib_) and parentage exclusion (*P*1, *P*2 and *P*3) probabilities were calculated with GenAlEx software (Peakall & Smouse, 2006); departures from Hardy-Weinberg equilibrium (HWE) at each locus and fixation index *F*is were calculated using the sofware FSTAT v2.9.3.2 (updated from Goudet (1995)). The presence of null alleles was tested with MICROCHECKER (Van Oosterhout et al., 2004). The Polymorphism Information Content (PIC) was estimated using CERVUS v3.0 (Kalinowski, Taper & Marshall, 2007).

## Results and Discussion

We identified a total of 106 polymorphic microsatellite loci (3 tri-, 103 tetranucleotides) across the seven species. Genetic variation was examined in 14–26 individuals per species. The number of polymophic loci per species varied between 14 and 17. A mean number of 6.4–8.3 alleles per locus were detected across the 106 loci in the seven species. Mean observed and expected heterozygosities ranged from 0.65 to 0.8 and 0.6 to 0.8, respectively. PIC values were relatively high, ranging between approximately 0.6 and 0.8 in all species. In some species, few loci exhibited departure from HWE (significant Fis) along with the presence of null alleles. Nevertheless, these results must be taken with caution since there is a high likelihood of close relatedness among samples (siblings, captive strains). Therefore, it is not possible to disentangle whether HWE departure results from inbreeding or null alleles. Identity and parentage exclusion probabilities were highly discriminant. In particular, the probability of identity *P*_IDsib_ (assuming the presence of siblings) varied from 1.1 × 10^−6^ to 3.3 × 10^−8^ across the seven species. Although based on a relatively small number of samples, these numbers are noteworthy given that most of our parrot individuals originate from aviaries and zoos and are expected to comprise full siblings or related individuals. Parentage exclusion probability when one parent is known (*P*1), when genotype of one parent is missing (*P*2) and of a pair of individuals as parents (*P*3) ranged from 0.995 to 1. Results overall loci and per locus are summarized in Tables 1 and S1, respectively.

**Table 1:** Characterization of polymorphic microsatellite loci by species in the seven studied parrot taxa (Psittacidae, Aves).

Species	n	No. loci	Mean N*A*	Mean *H*_O_	Mean *H*_E_	*Fis*	*P*_ID_	*P*_IDsib_	*P*1	*P*2	*P*3
*Amazona brasiliensis*	15	17	8.0	0.8	0.8	**0.076**	1.5 × 10^−21^	3.3 × 10^−8^	1.000000	0.999975	1.000000
*Amazona oratrix*	20	15	8.3	0.8	0.8	0	9.7 × 10^−20^	1.7 × 10^−7^	1.000000	0.999945	1.000000
*Amazona pretrei*	14	14	6.6	0.7	0.7	0.088	1.6 × 10^−13^	8.3 × 10^−6^	0.999929	0.995417	1.000000
*Amazona rhodocorytha*	19	14	7.8	0.8	0.8	−0.015	1.5 × 10^−16^	1.3 × 10^−6^	0.999997	0.999548	1.000000
*Anodorhynchus leari*	26	16	6.4	0.65	0.6	0.005	7.4 × 10^−14^	5.1 × 10^−6^	0.999935	0.995004	1.000000
*Ara rubrogenys*	22	15	6.7	0.7	0.7	**0.085**	2.4 × 10^−16^	1.1 × 10^−6^	0.999995	0.999264	1.000000
*Primolius couloni*	19	15	6.7	0.7	0.8	0.079	1.7 × 10^−16^	8.6 × 10^−7^	0.999996	0.999310	1.000000

**Notes:**

Bold values for *Fis* indicate significant (p < 0.05) departure from HWE after Bonferroni correction.

n, number of individuals analyzed; No. loci, number of loci; Mean N*A*, mean number of alleles over loci; Mean *H*_O_, mean observed heterozygosity over loci; Mean *H*_E_, mean expected heterozygosity over loci; *Fis*, fixation index; *P*_ID_, probability of identity; *P*_IDsib_, sibling probability of identity; *P*1, probability of parentage exclusion when one parent is known; *P*2, probability of parentage exclusion when genotype of one parent is missing; *P*3, probability of parentage exclusion of a pair of individuals as parents.

Without conclusive evidence it is difficult to prosecute illegal trade in wildlife. DNA analysis provides this evidence and can conclusively prove whether birds have or have not been captive bred. Despite its obvious conservation importance, this approach has been so far rarely applied. Ringler (2012) performed a sibship analysis with heterologous (i.e. not species-specific) microsatellite markers in two captive-bred endangered parrot species (*Amazona collaria* and *A. agilis*), and was able to identify groups of full siblings indicative of whole-nest poaching. In three forensic caseworks involving Australian black-cockatoos (*Calyptorhinchus* spp.), White et al. (2012) determined illegal harvesting and trade activities with paternity and individual identity testings. Importantly, the authors established a population genotype database composed of several hundred wild individuals, which is invaluable for statistical inference and methodological compliance (Linacre et al., 2011), but an extremely difficult task when dealing with rare and endangered Neotropical parrot taxa such as those included in the present study (their wild populations being mostly declining and composed by few thousand mature individuals at best; International Union for Conservation of Nature and Natural Resources, 2015). Finally, Dawnay et al. (2009) validated 28 microsatellite markers (both specific and heterologous) for forensic individual and parentage analyses based on the allele frequencies estimated in wild populations (nr. of individuals: 99–190) of six bird of prey species (families Accipitridae and Falconidae).

The data produced in this study has the potential to provide authorities with the ability to investigate suspected poachers and smugglers, investigating false parentage claims or establishing a link between trace evidence and an individual (e.g. in case of stolen birds, or when a bird is illegally transferred between different locations). In addition, these markers can be used to implement accurate assessments of relatedness among individuals, which is a crucial issue in captive programs when establishing optimal breeding protocols to preserve genetic variation and minimize inbreeding.

In summary, we have developed and characterized microsatellite markers for seven Neotropical parrots threatened with extinction. These markers are able to provide robust and highly discriminatory DNA forensic evidence for identification and parentage analyses. They can be used to detect illegal trade and captive laundering of wild birds, and more generally should promote population-level analyses and conservation efforts in these threatened species.

## Supplemental Information

10.7717/peerj.2416/supp-1 Supplemental Information 1 Characterization of 106 polymorphic microsatellite loci in the seven studied parrot taxa (Psittacidae, Aves). A, number of alleles; HO, observed heterozygosity; HE, expected heterozygosity; PIC, Polymorphism Information Content; Fis, fixation index; Pnull, probability of null alleles; Range (bp), allele range in base pairs; GenBank, GenBank Accession Number. Bold values for Fis indicate significant (p < 0.05) deviation from HWE after Bonferroni correction; ns, not significant; *p < 0.05.

## Additional Information and Declarations

### DNA Deposition

The following information was supplied regarding the deposition of DNA sequences: GenBank accession numbers: KX632370–KX632475.

### Data Deposition

The following information was supplied regarding data availability: The raw data has been supplied as Supplemental Dataset Files.
